# Malignant transformation of a mature teratoma of the adrenal gland: A rare case report and literature review: Erratum

**DOI:** 10.1097/MD.0000000000009104

**Published:** 2017-12-01

In the article, “Malignant transformation of a mature teratoma of the adrenal gland: A rare case report and literature review”,^[1]^ which appeared in Volume 96, Issue 45 of *Medicine*, Dr. Ailian Liu's degree should have appeared as PhD.
